# Asbestos Fiber in Root-Canal Treatment

**Published:** 1893-03

**Authors:** Edward C. Kirk


ARTICLE VI.

### ASBESTOS FIBER IN ROOT-CANAL TREATMENT.

The use of liquid or semi-fluid substances for root-canal dressings or fillings is rendered extremely difficult in the superior teeth without the assistance of some fibrous material which acts as a carrier or vehicle to overcome the effect of gravitation, and so secure the placing of the dressing fully to the apex. Cotton wool, silk, or lamb's wool, and in fact all of the fibers used in this connection, present the uniform objection of inviting the absorption of secretions through their porosity, and by reason of their organic origin of developing a tendency to putrefactive changes with resulting irritating effects on the pericemental membrane. For the past two years I have used with much satisfaction for the purpose under consideration, a long-fiber Canadian abestos, which is to be had from the dealers in abestos materials. It should be obtained in its native condition as rock abestos, not separated into the fine woolly condition by the mechanical processes used in preparing it for commercial purposes. The best variety occurs in irregular masses, deep emerald green in color, with a fine striated structure in which the fibers are in bundles from one-half to two inches in length. For use in pulp-chambers, the fiber is readily obtained from such a mass by holding it in the left hand and rubbing off a sufficient quantity in a fine silky condition by using the ball of the right thumb applied at right angles to the length of the fibrous mass. By rubbing it in this manner for a short time a flock of extremely fine fiber will be obtained, which can be twisted into a suitable wisp, having great tensile strength and a certain rigidity which enables one to readily carry it, even when moistened with a medicament, to the apex of the canal. The material described has certain marked advantages over any other fibrous material in common use for the purpose—viz, it is inorganic and undecom posable by any of the substances ordinarily used for pulp-canal treatment; it is unaffected by the ferment agents there present; it is by reason of its non-combustible character readily and instantly sterilized by passing it through an alcohol flame and heating it to redness; it makes a suitable and, unalterable vehicle for the application of tincture of iodin, potassium permanganate, nitrate of silver, sulphuric acid, or any of the agents which disintegrate cotton or wool fiber, as these are prone to do when used in concentrated solution. Further, it makes a most excellent vehicle in connection with oxychlorid of zinc, choro-percha, or paraffin as a permanent and unalterable root-filling material.—EDWARD C. KIRK, in *Cosmos.*

